# Phylogenetic analyses place the Australian monotypic *Revwattsia* in *Dryopteris* (Dryopteridaceae)

**DOI:** 10.3897/phytokeys.14.3446

**Published:** 2012-07-30

**Authors:** Meghan McKeown, Michael Sundue, David S. Barrington

**Affiliations:** 1 Pringle Herbarium, Department of Plant Biology, University of Vermont, 63 Carrigan Drive, Burlington Vermont, 05405 USA

**Keywords:** Biogeography, Australia, Morphology, Polystichum, Rumohra

## Abstract

*Revwattsia fragilis* (Watts) D.L. Jones (Dryopteridaceae), originally described as a *Polystichum* Roth by the pioneer Australian botanist Reverend W.W. Watts in 1914, is a rare epiphytic fern endemic to northeastern Queensland, Australia. Known from only a few populations, it is restricted to tropical rainforests in the Atherton Tablelands. We used the cpDNA markers *psbA-trnH*, *rbcL*, *rbcL-accD*, *rps4-trnS*, *trnG-trnR*, *trnL-trnF*, and *trnP-petG* to infer the relationships of *Revwattsia fragilis* within Dryopteridaceae. Based on our molecular analysis, we were able to reject Watts’s 1914 hypothesis of a close relationship to *Polystichum*. Its closest allies are a suite of Asian *Dryopteris* Adans. species including *Dryopteris labordei*, *Dryopteris gymnosora*, *Dryopteris erythrosora* and *Dryopteris cystolepidota*; maintaining *Revwattsia* renders *Dryopteris* paraphyletic. The epiphytic habit and distinctive long-creeping rhizome of *Revwattsia* appear to be autapomorphies and do not warrant its generic status. In the course of our investigation we confirmed that polyphyly of *Dryopteris* is also sustained by the inclusion of *Acrorumohra* (H.Itô) H.Itô, *Acrophorus* C.Presl, *Arachniodes* Blume, *Diacalpe* Blume, *Dryopsis* Holttum & P.J.Edwards, and *Peranema* D.Don. The epithet *fragilis* is occupied in *Dryopteris*, therefore we provide the name *Dryopteris wattsii* **nom. nov.** to accommodate *Revwattsia fragilis* in *Dryopteris*.

## Introduction

The fern genera *Polystichum* Roth and *Dryopteris* Adans. are now understood to be closely allied members of the Dryopteridaceae. *Polystichum* and its allies *Cyrtomium* C.Presl and *Phanerophlebia* C.Presl are sister to *Arachniodes* Blume and *Dryopteris* (Schuettpelz and Pryer 2007). The breadth of morphological diversity exhibited by *Polystichum* and *Dryopteris* has, in hindsight, had at least three impacts on the taxonomic history of these genera and their family. First, a large number of segregate genera have been removed from these two genera based on dramatic morphological transformations. Some of these segregates render *Dryopteris* and *Polystichum* paraphyletic; examples include *Sorolepidium* Christ, which belongs in *Polystichum* (Liu et al. 2007a), and *Lithostegia* Ching, which belongs in *Arachniodes* (Liu et al. 2007b). Second, these morphologically innovative lineages are sometimes superficially similar to (i.e. convergent with) remotely related ferns, leading to their circumscription as polyphyletic genera. For example, the morphologically anomalous *Polystichum speciosissimum* (Kunze) R.M.Tryon & A.F.Tryon was originally described in *Cheilanthes* Sw. (Pteridaceae). Third and central here, more remote members of the Dryopteridaceae superficially resemble species of *Polystichum* and *Dryopteris*. For instance, the epiphytic genus *Rumohra* Raddi was long included in *Polystichum*, presumably because of its peltate indusium (Diels 1902). However, Little and Barrington (2003) provided evidence for a close relationship of *Rumohra* to *Megalastrum* Holttumand *Lastreopsis* Ching, a conclusion confirmed in analyses with denser sampling more recently (Schuettpelz and Pryer 2007). This same relationship was implied by Tryon and Tryon (1982) who grouped *Rumohra*, *Megalastrum*, and *Lastreopsis* together in their key to dryopterid genera based on their shared central adaxial costal ridge.

**Figure 1.**
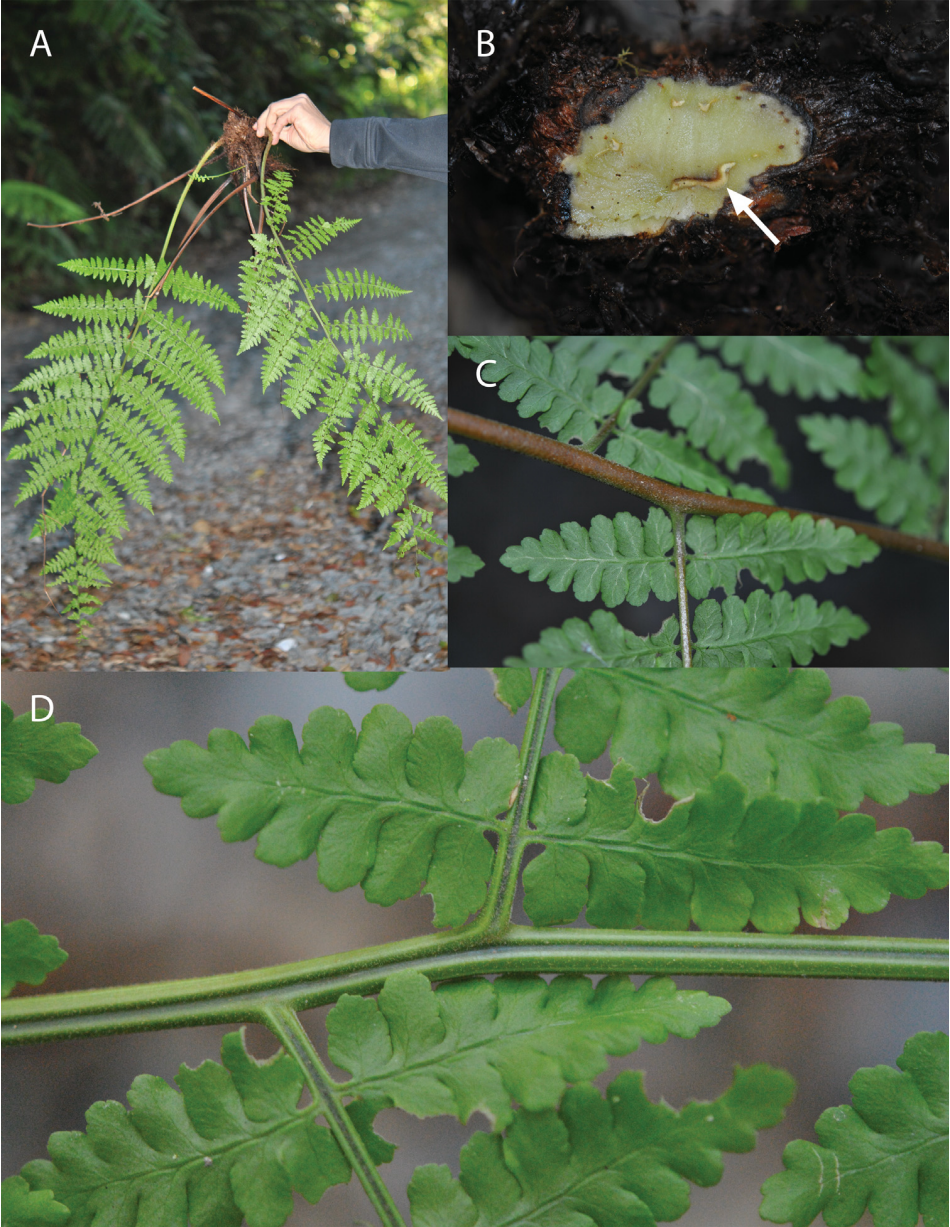
*Revwattsia fragilis*. **A** habit **B** Rhizome in cross section **C** Abaxial rachis and costa **D** Adaxial rachis and costa (M Kessler, M Sundue and M Lehnert 14293).

The rare Australian monotypic genus *Revwattsia* D.L.Jones presents a similarly intricate history (Fig. 1A). A high-canopy epiphyte, *Revwattsia fragilis* (Watts) D.L.Jones (Dryopteridaceae) is endemic to northeastern Queensland, where it is known from only a few small populations (Australia’s Virtual Herbarium 2012). *Revwattsia fragilis* is confined to mid-elevation rainforest, where it grows inside rotting tree hollows and among other epiphytes (Jones 1998). The Reverend W.W. Watts originally described *Revwattsia fragilis* in 1915 (‘1914’) as a *Polystichum*, presumably because of its perceived similarity to *Rumohra adiantiformis* (G. Forst.) Ching, which was then included in *Polystichum*. In northern Queensland, *Rumohra adiantiformis* is a common species in the humid forests; the two share a few superficial similarities: a long-creeping dorsiventral rhizome and epiphytic habit (Watts 1914)(Fig. 1B). Watts accurately listed characters by which *Revwattsia fragilis* differed from *Revwattsia adiantiformis*, including its reniform indusia, its less coriaceous texture, and lamina axes lacking a central adaxial costal ridge (Fig. 1D). Andrews (1990) and later Jones (1998) both emphasized what they perceived to be unique characters of *Revwattsia fragilis*. Andrews (1990) suggested recognition as a separate genus for the taxon in his treatment of the ferns of Queensland. Jones (1998) followed this lead in establishing the genus *Revwattsia* in his treatment to the Dryopteridaceae of Australia.

Indeed, inclusion of *Revwattsia* in *Polystichum* is untenable morphologically. Long-creeping rhizomes, reniform indusia, and the epiphytic habit are not characteristic of *Polystichum*. The herbaceous dark-brown petiole scales of *Revwattsia* are unknown in *Polystichum*, which has pale scales or dark indurated petiole scales. The extensive glandular indument characteristic of *Revwattsia* (Andrews 1990) is unknown among the mature fronds of large *Polystichum* species. In addition, the symmetrical ultimate segments are unknown in *Polystichum* species with large laminae. *Revwattsia* does, however, present morphological features suggestive of a relationship to *Dryopteris*, including the reniform indusium and capitate-glandular indument; characters which are common in *Dryopteris*. On the contrary, the long-creeping rhizome of *Revwattsia* is virtually unknown in *Dryopteris* (present in *Dryopteris amurensis* Christ and *Dryopteris angustifrons* (T. Moore) Kuntze), as is the epiphytic habit (known in the tropical American species *Dryopteris patula* (Sw.) Underw.). Furthermore, the dorsiventral rhizome is absent from the clade that includes *Polystichum* and *Dryopteris*.

*Revwattsia* presents a taxonomist’s classic dilemma; taxonomic placement requires a considered set of decisions about which morphological characters are synapomorphies and which are not. To address this dilemma, we assembled a set of chloroplast DNA nucleotide data from seven markers to infer the phylogenetic relationships of *Revwattsia* and provide insight into its morphological evolution. Included in our inquiry was a test of Jones’ 1998 assertion that *Revwattsia fragilis* requires a separate genus within the Dryopteridaceae. In order to understand implications of the taxonomic placement of *Revwattsia fragilis*, we also studied its critical morphological characters, namely those of the rhizome, indument, rachis and costa architecture, lamina segment shape, and indusium shape.

## Methods

### Material

*Revwattsia fragilis* was collected in the Cook District, Queensland, Australia, along the Mt. Lewis road, ca. 12 km before the shelter at the end of the rd. 16°36'S, 145°17'E, 900 m, M Kessler, M Sundue and M Lehnert 14293 (BRI, VT), 10 Aug 2011. Material for genetic analysis was stored in silica gel until DNA could be extracted. The permit used to collect this material was issued by Dept. of Environment and Resource Management Queensland (Michael Sundue, permit number WISP09438311).

### Morphology

Characters for *Revwattsia fragilis* were scored from M Kessler, M Sundue and M Lehnert 14293at The Pringle Herbarium (VT), and from previously published literature (Watts 1914, Andrews 1990, Jones 1998). We reviewed all salient features, but with particular attention to characters relevant to generic placement i.e. rhizome symmetry and morphology, rachis and costa architecture, lamina dissection, indument, and indusium shape.

### Taxon sampling

One-hundred and ninety-eight taxa from 36 genera were used in the phylogenetic analyses including 32 from the Dryopteridaceae. Taxonomic sampling was informed by an initial blast search of the *Revwattsia fragilis rbcL* sequence against the NCBI database (Altschul et al. 1990). The most similar *rbcL* sequences were *Dryopteris erythrosora* (D.C.Eaton) Kuntze,* Dryopteris cystolepidota* (Miq.) C.Chr.,and *Dryopteris championii* (Benth.) C.Chr., with 98.6% pairwise identity. Accordingly, our sampling was heaviest in *Dryopteris*, but also included a diverse selection of Dryopteridaceae. We also included more distant outgroups from the Lomariopsidaceae and Pteridaceae. As several generic segregates of *Dryopteris* are suspected to be nested within the genus (Liu et al. 2007b), we included accessions of *Acrorumohra* (H.Itô) H.Itô, *Acrophorus* C.Presl, *Arachniodes* Blume, *Diacalpe* Blume, *Dryopsis* Holttum & P.J.Edwards, *Nothoperanema* (Tagawa) Ching, and *Peranema* D.Don in this study. Some of these taxa have combinations in *Dryopteris*, however recent authors (Liu et al. 2007b, Wu 1999) have treated them under these alternate genera. We use the alternate names to highlight their phylogenetic position. Sequences other than those for *Revwattsia fragilis* were downloaded from GenBank; they are primarily from the work reported in Sessa et al. 2012 and Liu et al. 2007a (accession number and herbarium voucher information, Appendix 1).

### DNA extraction, amplification and sequencing

Total DNA extraction from silica-dried specimens was accomplished following the CTAB protocol of Doyle and Dickson (1987). Using the Techne TC3000 thermocycler (Techne, Duxford, UK) and the polymerase chain reaction (PCR), two intergenic spacers, *trnG-trnR* and *rps4-trnS*, were amplified for *Revwattsia fragilis*. The primers TRNG1F and TRNR22R (Nagalingum et al. 2007) were used to amplify *trnG-trnR*. Reactions were carried out in 25 mL volumes and included 2.5 mL of 10X PCR buffer, 0.5 mL of 10mM dNTPs, 0.5 mL of 100X BSA, 1.25 mL of the 10 mM forward primer, 1.25 mL of the 10mM reverse primer, 17.85 mL of ddH_2_O, 0.15 mL of Ex Taq Polymerase, and 0.5 mL of extracted DNA from *Revwattsia fragilis*. The thermocycler conditions for amplifying *trnG-trnR* comprised an initial denaturation of 2 minutes at 95°C followed by a core sequence of 35 repetitions of 95°C for 30 seconds, 45°C for 30 seconds, and 71°C for 1 minute followed by a final extension of 5 minutes at 71°C. The primers rps4-3er.f (Skog et al. 2004) and trnSr (Souza-Chies et al. 1997) were used to amplify *rps4-trnS*. Reaction conditions for *rps4-trnS* were the same as for *trnG-trnR*. Thermocycler conditions for amplifying *rps4-trnS* comprised an initial denaturation of 3 minutes at 94°C followed by 35 repetitions of 94°C for 1 minute, 55°C for 1 minute, and 72°C for 2 minutes followed by a final extension of 8 minutes at 72°C. *Revwattsia* rbcL sequences were generated following Schuettpelz and Pryer (2007) using the primers ESRBCL1F and ESRBCL1361R. Resulting PCR products were electrophoresed on a 1% agarose gel in 1x Tris-borate-EDTA (TBE) buffer (pH 8.0) containing ethidium bromide to visualize bands. Automated sequencing took place on an ABI Prism 3130x1 sequencer at the Vermont Cancer Center, Burlington, Vermont, USA. Sequencing primers for *rps4-trnS* were the same primers used for the template amplification. For *trnG-trnR* analysis we used the following sequencing primers: TRNG1F. TRNR22R, TRNG43F, and TRNG63R (Nagalingum et al. 2007). For *rbcL* sequencing we used the amplification primers in addition to ESRBCL628F and ESRBCL654R (Schuettpelz and Pryer 2007).

### Sequence alignment and coding

Sequences were edited and aligned using Geneious v5.4.2 (Drummond et al. 2011) and then manually checked for errors. Markers were analyzed separately using Modeltest v3.06 (Posada and Crandall 1998) to determine the model of evolution that each marker most closely fit (Table 1) using the Akaike information criterion (AIC). Indels were coded using the program SeqState 1.4.1 (Müller 2005) and treated in the matrix as standard data.

**Table 1.** Characteristics of the cpDNA markers used in the phylogenetic analyses.

**Marker**	**Model (AIC)**	**Aligned Length of Marker**	**% Parsimony Informative**	**Taxa sampled**
rbcL	SYM+I+G (26503)	1506	19%	194
trnG-trnR	TIM+I+G (16303)	1290	40%	100
pbsA-trnH	TVM+G (4308)	584	30%	101
rbcL-accD	GTR+I+G (9942)	961	37%	99
trnL-trnF	GTR+G (4594)	297	57%	102
rps4-trnS	TVM+G (7704)	576	51%	101
trnP-petG	TIM+G (9370)	623	50%	99

### Phylogenetic analyses

Bayesian inference was conducted on the concatenated data set (*psbA-trnH*, *rbcL*, *rbcL-accD*, *rps4-trnS*, *trnG-trnR*, *trnL-trnF*, and *trnP-petG*) using MrBayes v3.2.0 (Ronquist et al. 2011) using the appropriate evolutionary models determined for each. Sampling of all seven loci was primarily within *Dryopteris*; the remaining taxa, including *Revwattsia fragilis*, had subsets of the seven loci. The Markov chain Monte Carlo permutation of tree parameters was conducted for 2 runs of 5,000,000 generations, sampling every 100th generation. A plot of generations versus log-likelihood was examined using Tracer v1.5 (Rambaut and Drummond 2009) to visually assess stationarity and verify that an appropriate burn-in was achieved. The burn-in was 500,000 generations. The 50% majority-rule tree was examined in FigTree v1.3.1 (Rambaut 2009).

Parsimony analyses using the same data set were conducted using TNT (Willi Hennig Society, Goloboff et al. 2008) implementing the parsimony ratchet (Nixon 1999), with the following search parameters: 1000 ratchets with 200 iterations per replicate, 10% weighting, holding 20 trees per ratchet, followed by tree-bisection-reconnection (TBR) branch swapping to completion. Clade support was assessed by implementing a bootstrap analysis of 1000 replicates with 10 ratchets per replicate and holding 20 trees per ratchet. The max RAM was set at 850 MB allowing for storage of 10,000 trees.

## Results

### Phylogenetic analyses

**Figure 2.**
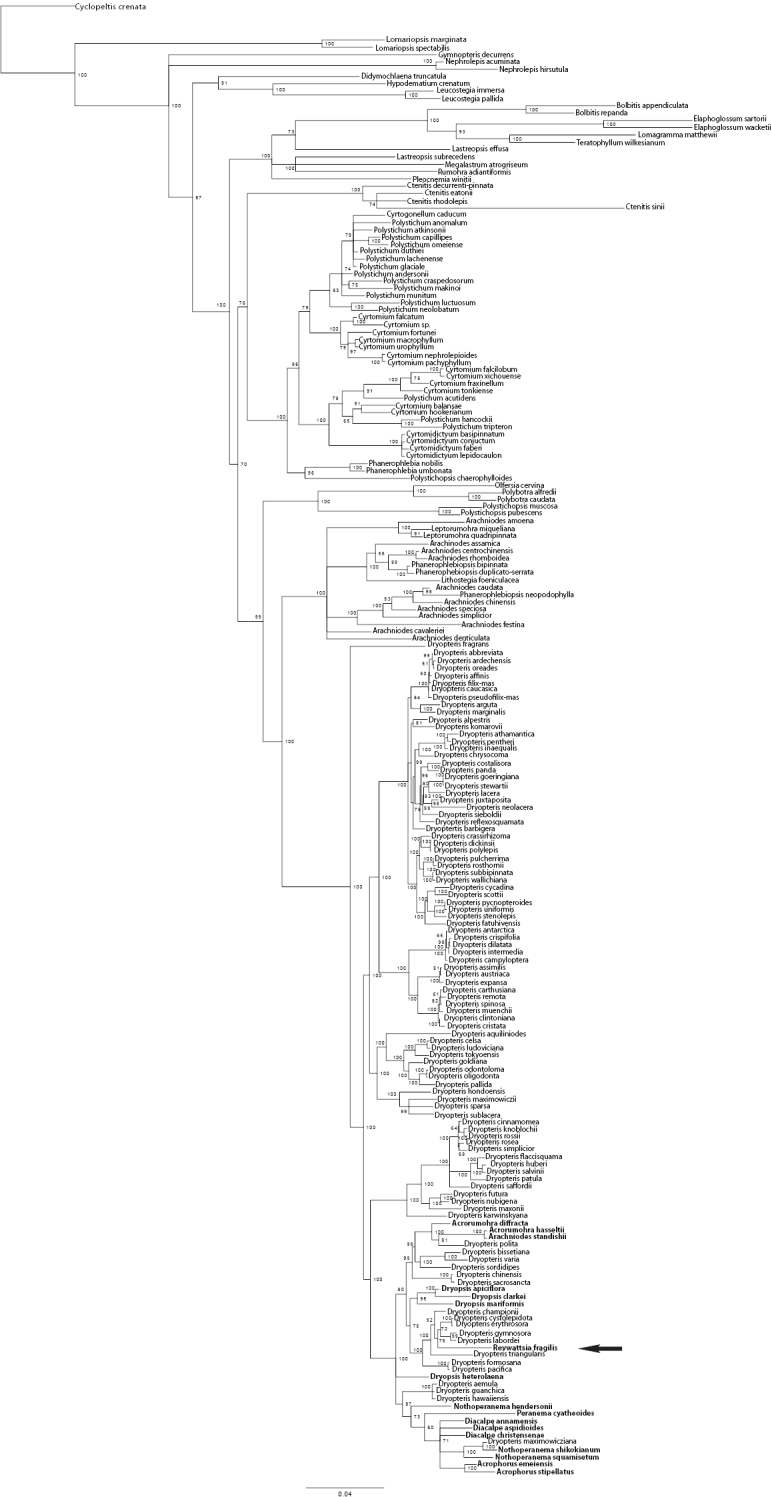
The 50% majority rule tree resulting from Bayesian analysis. Values indicate posterior probabilities, scale bar indicates 0.04 substitutions per site. Arrow indicates position of Revwattsia fragilis.

**Figure 3.**
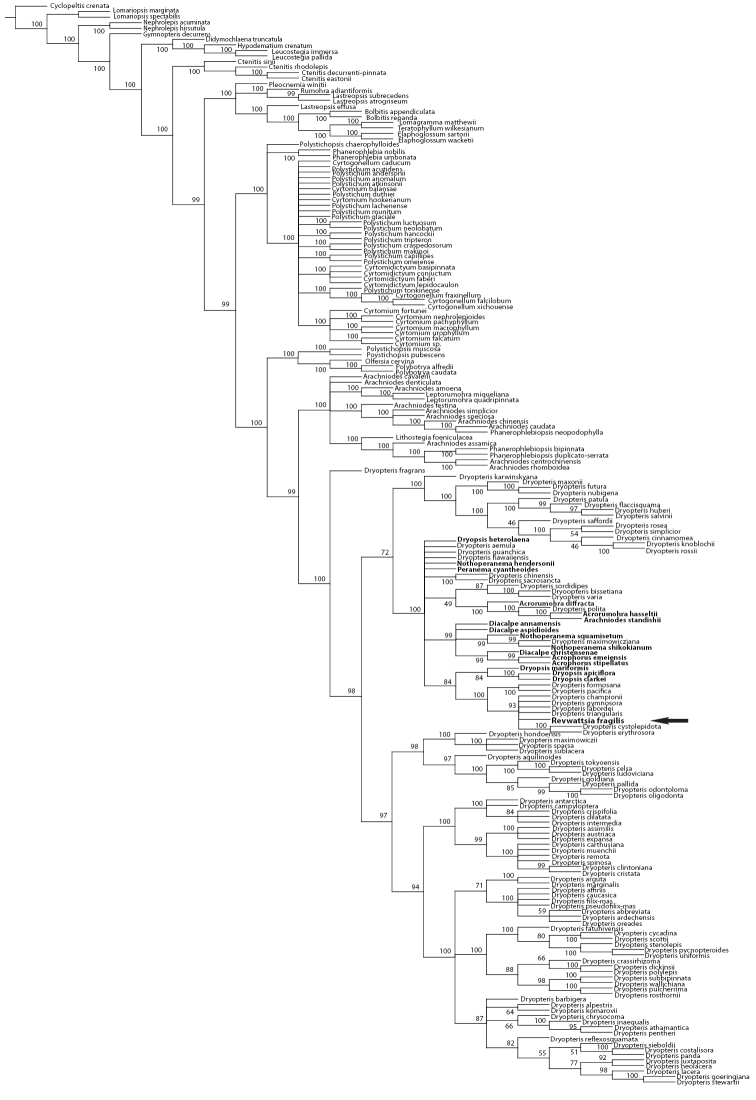
Strict consensus of 10,000 most parsimonious trees. Values indicate bootstrap support of 1000 pseudoreplicates. Arrow indicates position of Revwattsia fragilis.

Of the 5425 total characters, 1717 characters (31.6%) were parsimony informative. In the maximum parsimony analysis (MP) 10,000 most parsimonious trees were retained before maximum storage capacity was reached. The shortest trees had a length of 5531 steps, a consistency index (CI) of 0.40, and retention index (RI) of 0.79. The topology of the Bayesian inference (BI) 50% majority rule tree was largely congruent with the topology of the MP tree but allowed greater resolution of the taxa allied to *Revwattsia fragilis*. Results of the BI and MP analyses place *Revwattsia fragilis* in a recently diverged clade within the genus *Dryopteris* (Figures 2 and 3).

In both analyses, there is strong support for placement of *Revwattsia fragilis* within a clade of *Dryopteris* comprising species from southern and eastern Asia. In the Bayesian analysis, *Revwattsia fragilis* is sister to the clade comprising *Dryopteris cystolepidota*, *Dryopteris erythrosora*, *Dryopteris gymnosora* (Makino) C.Chr., and *Dryopteris labordei* (Christ) C.Chr. (78% posterior probability). This clade in turn is sister to *Dryopteris championii* (92% posterior probability), followed by *Dryopteris triangularis* Herter(100% posterior probability). These same taxa form a clade in the MP analyses (93% bootstrap support), but relationships between these taxa collapse in the strict consensus of all most parsimonious trees.

### Morphological assessment

*Revwattsia fragilis* exhibits a massive (3 cm diam.) long-creeping rhizome with dorsal leaves and ventral roots (Figure 1A). A rhizome cross-section revealed an elongate ventral meristele (Figure 1B arrow). The rhizome and basal petiole are densely provided with thin, dark brown attenuate scales. The rachis and costa are rounded abaxially (Figure 1C), and are shallowly grooved adaxially (Figure 1D). The grooves are shallowly continuous with the next-order axis (Figure 1D) and they lack a central ridge. These axes are densely provided with short capitate-glandular hairs (Figure 1D). Frond dissection is 2-pinnate-pinnatifid to 2-pinnate-pinnatisect with symmetrical (neither basiscopically nor acroscopically enlarged) pinnae and pinnules (Figure 1A). Fertile fronds have medial sori and light brown reniform indusia.

## Discussion

### Monophyly of *Dryopteris*

Results presented here demonstrate that the monotypic genus *Revwattsia* is nested within *Dryopteris* (Figures 2 and 3). Maintaining *Revwattsia* renders *Dryopteris* paraphyletic; we therefore recommend placing the monotypic *Revwattsia* in synonymy under *Dryopteris*.

Paraphyly of *Dryopteris* is further perpetuated by the inclusion of the sampled *Acrophorus* (two species), *Acrorumohra* (two species), *Arachniodes standishii* (T. Moore) Ohwi, *Diacalpe* (three species), *Dryopsis* (three species),*Nothoperanema* (three species), and *Peranema cyatheoides* D. Don. These results do not come as a surprise given the results of other recent phylogenetic studies (Liu et al. 2007b, Geiger and Ranker 2005). The paraphyly of *Dryopteris* presented here corroborates long-standing suspicion about the circumscription of *Dryopteris* segregate genera (Tryon and Tryon 1982) and underscores the need for rich taxon sampling, particularly from Asia, in studies of Dryopteridaceae.

### Evolutionary implications

Our assessment of morphological characters largely corroborates those of Watts (1914), Andrews (1990), and Jones (1998). Most of the characters displayed by *Revwattsia fragilis* are known to occur within *Dryopteris*. The dark brown attenuate scales and capitate glandular hairs seen in *Revwattsia fragilis* occur frequently in *Dryopteris* (Kramer et al. 1990). The grooved rachis and costae are also typical of *Dryopteris* and many other dryopterid ferns (Holttum 1960). A reniform indusium is characteristic of most Dryopteridaceae and occurs throughout *Dryopteris* as it is currently circumscribed (other indusial shapes, which we take to be autapomorphies, are known from *Acrophorus*, *Diacalpe*, *Notheperanema*, and *Peranema*).

The long-creeping rhizome and elongate ventral meristele of *Revwattsia* (the latter first demonstrated here, Fig. 1B) are distinctive autapomorphies. Although a long-creeping rhizome is known to occur in *Dryopteris amurensis* and *Dryopteris angustifrons*, neither is closely allied to *Revwattsia fragilis*. These two characters occur in combination sporadically in Eupolypods I (e.g., in *Lomariopsis* Fée (Holttum 1978), the Bolbitidoid clade (Moran et al. 2010), and *Rumohra* (Kato 1974)) and appear to have evolved multiple times. In our experience this combination of characters appears to be correlated with strong dorsiventrality of the rhizome. We take this convergence between our subject species and *Rumohra adiantiformis*, the plant to which Watts presumably thought it most closely related, to be coincidental; Watts never cited these characters in his protolog.

### Biogeographic implications

Biogeographic patterns in *Dryopteris* were recently examined by Sessa et al. (2012)—however patterns among Australian taxa were not explicitly addressed. In addition to *Revwattsia fragilis*, Australia is home to three species of *Dryopteris*—*Dryopteris atrata* (Wall.) Ching, *Dryopteris cycadina* (Franch. & Sav.) C.Chr.,* D. sparsa* (D.Don) Kuntze (Jones 2012)—and *Acrorumohra hasseltii* (Blume) Ching. All but *Dryopteris atrata* are included in our analysis. Unlike *Revwattsia fragilis*, these species have relatively broad ranges including India and Sri Lanka, southern China and Japan, and Malesia. In each of these cases, the closest relatives are distributed in southern and eastern Asia, suggesting this region as ancestral for each of the Australian taxa. These species are resolved in clades distinct from each other and from *Revwattsia fragilis*, indicating that at least four separate migration events are necessary to explain the current distribution of *Dryopteris* (including *Revwattsia fragilis* and *Acrorumohra hasseltii*) in Australia. The inclusion of the unsampled *Dryopteris atrata* in future studies may increase the inferred number of migrations. Our results are comparable to those of Li et al. (2007), who revealed similar migration events from Southern Asia to Australia in the closely related genus *Polystichum*.Although the Sunda and Sahul shelves are currently divided by a deep oceanic trench, these regions were in close proximity 23 mya during the time of the divergence of *Dryopteris* (Sessa et al. 2012, Lohman et al. 2011). It remains unclear whether the migration of *Dryopteris* can be attributed to long distance dispersal or incremental range expansion.

### Circumscription of *Dryopteris*

The phylogenetic position of species treated as *Acrophorus*, *Acrorumohra*, *Arachniodes standishii*, *Dryopsis*, *Nothoperanema*, *Peranema*, and *Revwattsia fragilis* demonstrate that the circumscription of *Dryopteris* needs to be expanded. Several of these genera include unique character states that do not occur in *Dryopteris* as currently defined. In addition to the morphological redefinition, expansion of *Dryopteris* to include these segregate genera necessitates numerous nomenclatural innovations. We provide here a name for *Revwattsia fragilis* in *Dryopteris*. The name *Dryopteris fragilis* is previously occupied; therefore a new name is provided.

### Taxonomy and nomenclature

***Dryopteris wattsii***, M. McKeown, Sundue, & Barrington nom. nov. ≡ *Polystichum fragile* Watts, Proc. Linn. Soc. New South Wales 39: 775. 1914 (1915). ≡ *Revwattsia fragilis* (Watts) D.L.Jones [as “*Revwattsia fragile*”], Flora of Australia 48:711. 1998. non *Dryopteris fragilis* C. Chr. TYPE: Australia, Queensland, Majors Homestead, near Ravenshoe, W. W. Watts s.n., Aug 1913 (Syntypes: BRI n.v., MEL n.v., NSW n.v.).

## Appendix

GenBank accession numbers for McKeown et al. 2012 (doi: 10.3897/phytokeys.14.3446.app) File format: Adobe PDF file (PDF).

**Explanation note:** Genbank Accession numbers are listed in the following order: psbA-trnH, rbcL, rbcL-accD, rps4-trnS, trnG-trnR, trnL-trnF, trnP-petG. The “—“ indicates markers that were not available for the taxon.

## References

[B1] Altschul S Gish W Miller W Myers E Lipman D (1990) Basic local alignment search tool. Journal of Molecular Biology 215 (3): 403–410. 10.1016/S0022-2836(05)80360-2 2231712

[B2] Andrews SB (1990) Ferns of Queensland. Queensland Department of Primary Industries. Brisbane.

[B3] Australia’s Virtual Herbarium (2009). [map output], Council of Heads of Australasian Herbaria, viewed 23 May 2012, http://www.chah.gov.au/avh/.

[B4] Diels L (1902) Polystichum. In: Engler A, Prantl K. Die natürlichen Pflanzenfamilien. I. Teil. Abteilung 4. Wilhelm Engelmann, Leipzig, 189–194.

[B5] Doyle JJ Dickson EE (1987) Preservation of plant-samples for DNA restriction endonuclease. Taxon 36 (4): 715-722 doi: 10.2307/1221122

[B6] Drummond AJ Ashton B Buxton S Cheung M Cooper A Duran C Field M Heled J Kearse M Markowitz S Moir R Stones-Havas S Sturrock S Thierer T Wilson A (2011) Geneious v5.4, Available from http://www.geneious.com

[B7] Geiger JMO Ranker TA (2005) Molecular phylogenetics and historical biogeography of Hawaiian *Dryopteris* (Dryopteridaceae). Molecular Phylogenetics and Evolution 34: 392-407 doi: 10.1016/j.ympev.2004.11.001 15619450 10.1016/j.ympev.2004.11.001

[B8] Goloboff PA Farris JS Nixon KC (2008) TNT, a free program for phylogenetic analysis. Cladistics 24 (5): 774-786 doi: 10.1111/j.1096-0031.2008.00217.x

[B9] Holttum RE (1978)*Lomariopsis* group. In: van Steenis GGJ, Holttum RE, eds. Pteridophyta, ferns and fern allies. Flora Malesiana Series II, vol 1, pt 4., 255–330.

[B10] Holttum RE (1960) Vegetative characters distinguishing the various groups of ferns included in Dryopteris of Christensen’s Index Filicum, and other ferns of similar habit and sori. Gardens’ Bulletin, Singapore 17: 361-367

[B11] Jones DL (1998) Flora of Australia, Ferns, Gynosperms and Allied Groups.Vol. 48, Melbourne: ABRS/CSIRO Australia

[B12] Jones DL (2012) *Dryopteris*. Flora of Australia Online. Australian Biological Resources Study, Canberra. Viewed 24 May 2012. http://www.environment.gov.au/biodiversity/abrs/online-resources/flora/main/index.html

[B13] Kato M (1974) A note on the systematic position of *Rumohra adiantiformis*. Acta Phytotaxonomica et Geobotanica 26: 123-158

[B14] Kramer KU Holttum RE Moran RC Smith AR (1990) Dryopteridaceae, in Kramer KU, Green PS (1990) 1. Pteridophytes and Gymnosperms. In: Kubitzki K (Ed) The families and genera of vascular plants. Springer-Verlag, Berlin.

[B15] Li CX Lu SG Yang Q (2007) Phylogeny and biogeography of Chinese and Australasian *Polystichum* ferns as inferred from chloroplast *trnL-F* and *rps4-trnS* sequence data. Palaeoworld 16 (4): 294-300 doi: 10.1016/j.palwor.2007.07.003

[B16] Little DP Barrington DS (2003) Major evolutionary events in the origin and diversification of the fern genus *Polystichum* (Dryopteridaceae). American Journal of Botany 90 (3): 508-514 doi: 10.3732/ajb.90.3.508 21659143 10.3732/ajb.90.3.508

[B17] Liu HM Zhang XC Chen ZD Qiu YL (2007a) Inclusion of the Eastern Asia endemic genus *Sorolepidium* in *Polystichum* (Dryopteridaceae): evidence from the chloroplast rbcL gene and morphological characteristics. Chinese Science Bulletin 52 (5): 631-638 doi: 10.1007/s11434-007-0115-2

[B18] Liu HM Zhang XC Wang W Qui YL Chen ZD (2007b) Molecular phylogeny of the fern family Dryopteridaceae inferred from chloroplast *rbcL* and *atpB* genes. International Journal of Plant Science 168 (9): 1311-1323 doi: 10.1086/521710

[B19] Lohman DJ Bruyn M Page T von Rintelen K Hall R Ng PKL Shih HT Carvalho GR von Rintelen T (2011) Biogeography of the Indo-Australian Archipelago. Annual Review of Ecology, Evolution, and Systematics 42: 205-226 doi: 10.1146/annurev-ecolsys-102710-145001

[B20] Nagalingum NS Schneider H Pryer KM (2007) Molecular phylogenetic relationships and morphological evolution in the heterosporous fern genus *Marsilea*. Systematic Botany 32 (1): 16-25 doi: 10.1600/036364407780360256

[B21] Moran RC Labiak PH Sundue MA (2010) Phylogeny and character evolution of the bolbitidoid ferns (Dropteridaceae). International Journal of Plant Science 171: 547-549 doi: 10.1086/652191

[B22] Müller K (2005) SeqState - primer design and sequence statistics for phylogenetic DNA data sets. Applied Bioinformatics 4: 65-69 16000015 10.2165/00822942-200504010-00008

[B23] Nixon KC (1999) The Parsimony Ratchet, a new method for rapid parsimony analysis. Cladistics 15(4): p. 407–414. doi: 10.1111/j.1096-0031.1999.tb00277.x 10.1111/j.1096-0031.1999.tb00277.x 34902938

[B24] Posada D Crandall KA (1998) Modeltest: testing the model of DNA substitution. Bioinformatics 14 (9): 817-818 doi: 10.1093/bioinformatics/14.9.817 9918953 10.1093/bioinformatics/14.9.817

[B25] Rambaut A (2009) FigTree v1.3.1 http://tree.bio.ed.ac.uk/software/figtree

[B26] Rambaut A Drummond AJ (2009) Tracer *v1.5*. http://tree.bio.ed.ac.uk/software/tracer

[B27] Ronquist F Huelsenbeck JP (2003) MrBayes 3: Bayesian phylogenetic inference under mixed models. Bioinformatics 19: 1572-1574 doi: 10.1093/bioinformatics/btg180 12912839 10.1093/bioinformatics/btg180

[B28] Sessa EB Zimmer EA Givnish TJ (2012) Phylogeny, divergence times, and historical biogeography of New World *Dryopteris* (Dryopteridaceae). American Journal of Botany 99 (4): 730-750 doi: 10.3732/ajb.1100294 22434775 10.3732/ajb.1100294

[B29] Schuettpeltz E Pryer KM (2007) Fern phylogeny inferred from 400 leptosporangiate species and three plastid genes. Taxon 56 (4): 1037-1050 doi: 10.2307/25065903

[B30] Skog JE Mickel JT Moran RC Volovsek M Zimmer EA (2004) Molecular studies of representative species in the fern genus *Elaphoglossum* (Dryopteridaceae) based on cpDNA sequences rbcL,trnL-F , and rps4-trnS. International Journal of Plant Sciences 165: 1063-1075 doi: 10.1086/423877

[B31] Souza-Chies TT Bittar G Nadot S Carter L Besin E Lejeune B (1997) Phylogenetic analysis of Iridaceae with parsimony and distance methods using the plastid gene rps4. Plant Systematics and Evolution 204: 109–123. doi: 10.1007/BF00982535

[B32] Tryon R Tryon AF (1982) Additional taxonomic and nomenclatural notes on ferns. Rhodora 84 (837): 125-130

[B33] Watts WW (1915) [‘1914’] Some notes on the ferns of north Queensland. Proceedings of the Linnean Society of New South Wales Series 2, 39: 775, t. lxxxviii, fig. 9A–G.

[B34] Wu SH (1999) Peranemaceae. In: Wu CY (Ed). Flora Reipublicae Popularis Sinicae, vol.4(2). Science Press, Beijing: 216-238

